# Evaluating the effectiveness and roadside noise of alternative transverse rumble strip designs

**DOI:** 10.1038/s41598-026-48504-4

**Published:** 2026-05-19

**Authors:** Omar Sallam, Khaled El-Rayes, Ernest-John Ignacio, Ramez Hajj, Omar Almasry, Ahmed A. Hassan, Mamdouh Al-Ghzawi

**Affiliations:** Department of Civil and Environmental Engineering, The Grainger College of Engineering, University of Illinois Urbana-Champaign, Urbana, IL 61801 USA

**Keywords:** Transverse rumble strips, Rumble strip external noise, Rumble strip effectiveness, Traffic noise mitigation, Roadside noise, Roadway safety, Accident prevention, Engineering, Mathematics and computing

## Abstract

This study conducted field measurements to evaluate the performance of five promising traditional and alternative transverse rumble strip designs in generating effective in-vehicle noise level increases to alert distracted and drowsy drivers while reducing roadside noise levels. The five transverse rumble strip designs consisted of one baseline traditional design and four promising designs including shorter panel, angled, staggered, and sinusoidal designs. The in-vehicle and external noise measurements were conducted following the SAE J1477 standard and the AASHTO Statistical Isolated Pass-by (SIP) method, respectively. The noise measurements were collected using thirteen different vehicles with variations in type, make, size, weight, engine, and model including gasoline, hybrid, and electric sedans as well as SUVs, minivan, pick-up, box, and heavy semi-trailer trucks to represent all vehicles on U.S. roads. The findings of this study confirmed that all five transverse rumble strip designs generated average in-vehicle noise level increases between 3 and 15 dBA that fulfill the NCHRP recommendations for alerting distracted drivers. The analysis results also indicated that the four promising transverse rumble strip designs reduced roadside noise levels by a range of 18% (1.8 dBA) to 77% (7.7 dBA) compared to the baseline traditional design. These research findings provide state Departments of Transportation (DOTs) with new effective designs that maintain the roadway safety benefits of transverse rumble strips while decreasing their roadside noise levels and related complaints from nearby residents.

**Supplementary Information:**

The online version contains supplementary material available at 10.1038/s41598-026-48504-4.

## Background

Transverse rumble strips are reported to improve roadway safety by generating noise inside traveling vehicles to alert distracted and/or speeding drivers of the need to reduce their speed as they approach stop-controlled intersections, construction zones, or sharp curves^1–8^. For example, installation of transverse rumble strips before intersection were reported to reduce the speed of traveling vehicle by 1 mph to 5 mph^9–11^. Studies reported that installation of transverse rumble strips before intersections can reduce crash rates by 20% to 30%^12–14^. Accordingly, the National Cooperative Highway Research Program (NCHRP) has recommended an in-vehicle noise level increase range of 3 to 15 dBA above ambient to ensure the effectiveness of rumble strips in alerting distracted and drowsy drivers^15,16^. Despite the roadway safety benefits of transverse rumble strips, they were also reported to produce roadside noise that disturb nearby residents^12,17–21^. Studies have reported that regular exposure to traffic noise causes several negative effects on public health such as sleep disturbance and annoyance^22–25^.

To overcome these negative health effects of transverse rumble strips, few studies have evaluated the effectiveness and external noise performance of a limited number of transverse rumble strip designs to identify designs that can generate adequate in-vehicle noise levels to alert distracted drivers while reducing generated external noise and potential complaints from nearby residents. For example, Horne et al.^12^ utilized a passenger vehicle to measure and compare the external noise levels produced by two transverse rumble strip designs and reported that an epoxy-filled design reduced external noise by 5 dBA compared to the original traditional design. Hallmark et al.^6^ utilized a passenger vehicle to evaluate the effectiveness and the external noise generated by transverse rumble strips with alternative number of panels and rumble strips per panel. The findings of their study revealed that a transverse design with fewer rumble strips per panel slightly minimized external noise levels while still generating effective in-vehicle noise levels to warn distracted drivers. Elghamrawy et al.^26^ conducted field experiments using a sedan, a van, and a box truck to evaluate and compare the effectiveness of various layouts of temporary transverse rumble strips with different rumble strip cross sections, number of strips per panel, and strip spacing. They reported that the effectiveness of transverse rumble strips can be enhanced by increasing the number of strips per panel and using wider strips. Similarly, An et al.^27^ used three vehicles (a sedan, an SUV, and a dump truck) to collect and evaluate both the external and in-vehicle noise levels produced by four transverse rumble strip designs with different rectangular and rounded cross sections. The findings of their study showed that the transverse rumble strip design with rounded cross sections generated the lowest external noise levels compared to other designs while all four designs generated noticeable in-vehicle noise levels to alert drivers.

Despite the contributions of these studies, they evaluated the external and in-vehicle noise performances of limited variations of transverse rumble strip designs using a limited set of test vehicles. To overcome these limitations, there is a need to evaluate the effectiveness and external noise performance of various traditional and alternative transverse rumble strip designs using a representative sample of all vehicle sizes and types on U.S. roadways including varying sizes of gasoline, electric, and hybrid vehicles. This is expected to provide state Departments of Transportation (DOTs) with new knowledge on promising transvers rumble strip designs that are capable of generating adequate in-vehicle noise levels to alert distracted drivers and maintain their roadway safety while mitigating the generated roadside noise and its related complaints from nearby residents.

## Objective and methodology

The objective of this study is to conduct a comprehensive field study to evaluate the in-vehicle and external noise performance of five promising traditional and alternative transverse rumble strip designs that are expected to generate effective in-vehicle noise level increases to alert distracted and drowsy drivers while reducing roadside noise levels. The performance evaluation of these five promising designs was conducted using thirteen different vehicle types and sizes including sedan cars, SUVs, minivan, pickup trucks, box trucks, and heavy semi-trailer truck. To achieve this objective, a methodology of three phases was developed that focused on (1) identifying and constructing five promising transverse rumble strip designs including traditional, angled, staggered, and sinusoidal designs; (2) conducting noise measurements to collect in-vehicle and external noise levels generated by these five designs using thirteen test vehicles that represent all vehicle types and sizes on U.S. roads; and (3) analyzing the collected in-vehicle and external noise data to identify top-performing rumble strip designs that can generate in-vehicle noise increases between 3 and 15 dBA, as recommended by the NCHRP to alert distracted drivers while minimizing roadside noise and potential of related complaints from nearby residents.

### Identification and construction of transverse rumble strip designs

**Fig. 1:**
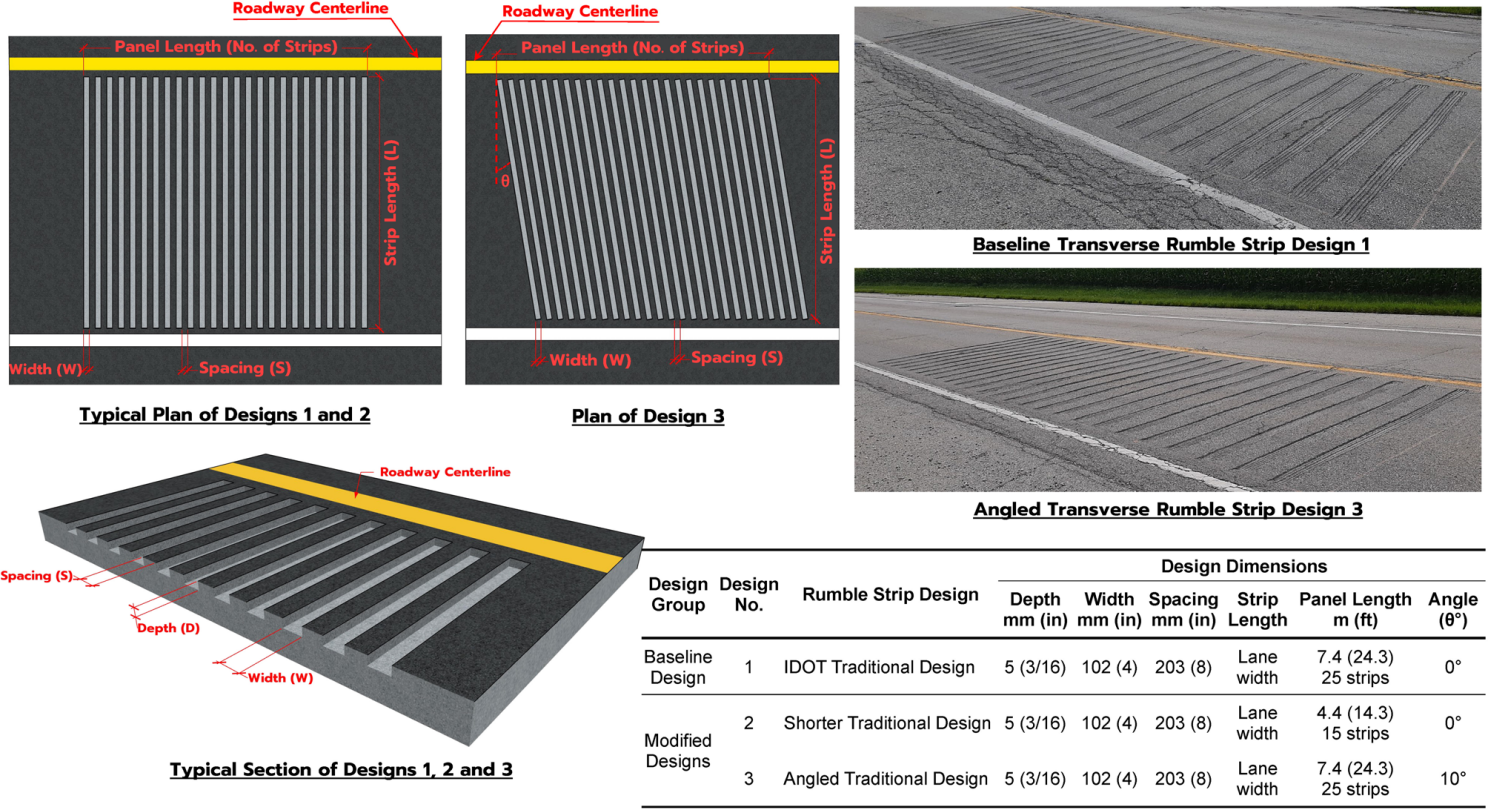
Baseline and modified traditional transverse rumble strip designs.

**Fig. 2:**
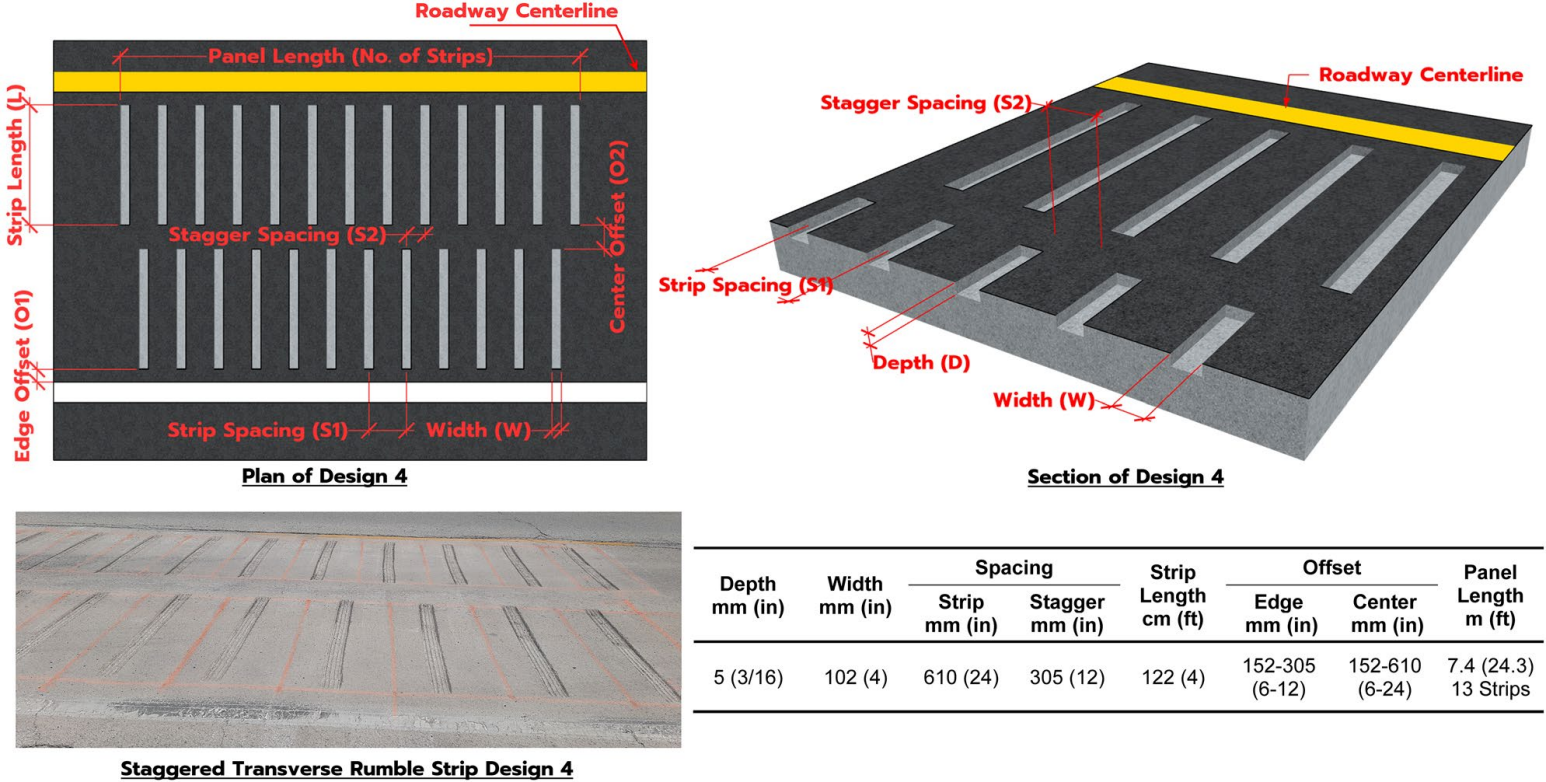
Staggered transverse rumble strip design.

**Fig. 3:**
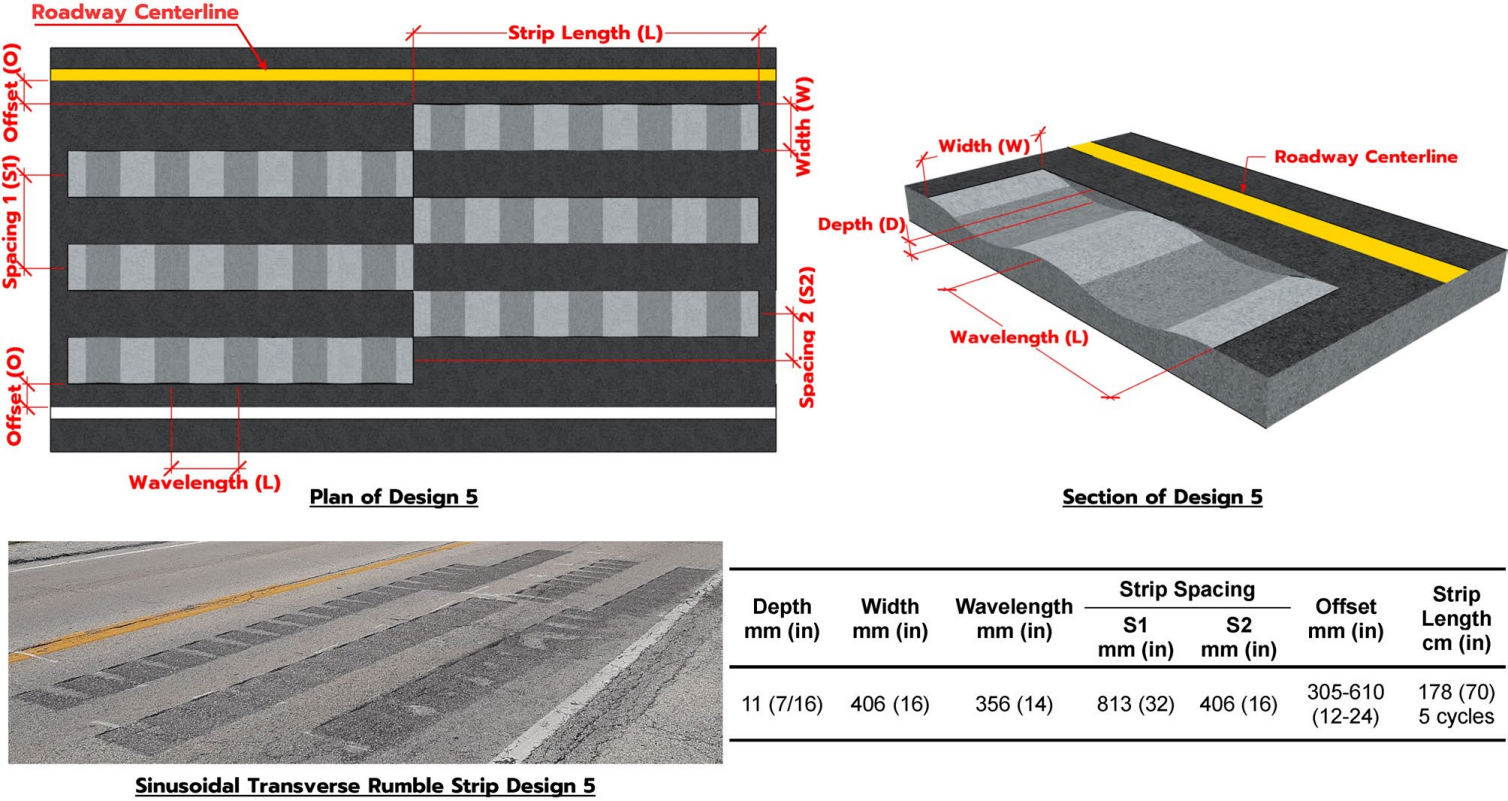
Sinusoidal Transverse rumble strip design.

Five promising traditional and alternative transverse rumble strip designs were identified and constructed to evaluate their effectiveness in generating sufficient in-vehicle noise level increases to alert distracted drivers while reducing roadside noise. These five transverse rumble strip designs consist of three groups. The first group includes one traditional transverse rumble design (Design 1) that was obtained from IDOT local roads and streets design manual and standards, as illustrated in Fig. 1. This traditional transverse design (Design 1) will be used as a baseline for evaluating the effectiveness and external noise performance of the remaining four designs. The second group of designs includes two promising modified transverse rumble strip designs (Designs 2 and 3) that provide new variations in design dimensions and parameters including shorter panel (Design 2) and angled transverse rumble strips (Design 3), as illustrated in Fig. 1. The third group of designs includes two promising alternative transverse rumble strip designs (Designs 4 and 5) that were designed to provide new geometric patterns of transverse rumble strips including staggered traditional transverse rumble strips (Design 4) and sinusoidal transverse rumble strips (Design 5), as shown in Figs. 2 and 3, respectively. These four promising designs were identified based on a comprehensive review of related literature and an analysis of previous studies that focused on mitigating roadside noise generated by rumble strips while maintaining their effectiveness in alerting inattentive drivers^6,12,27^.

**Fig. 4:**
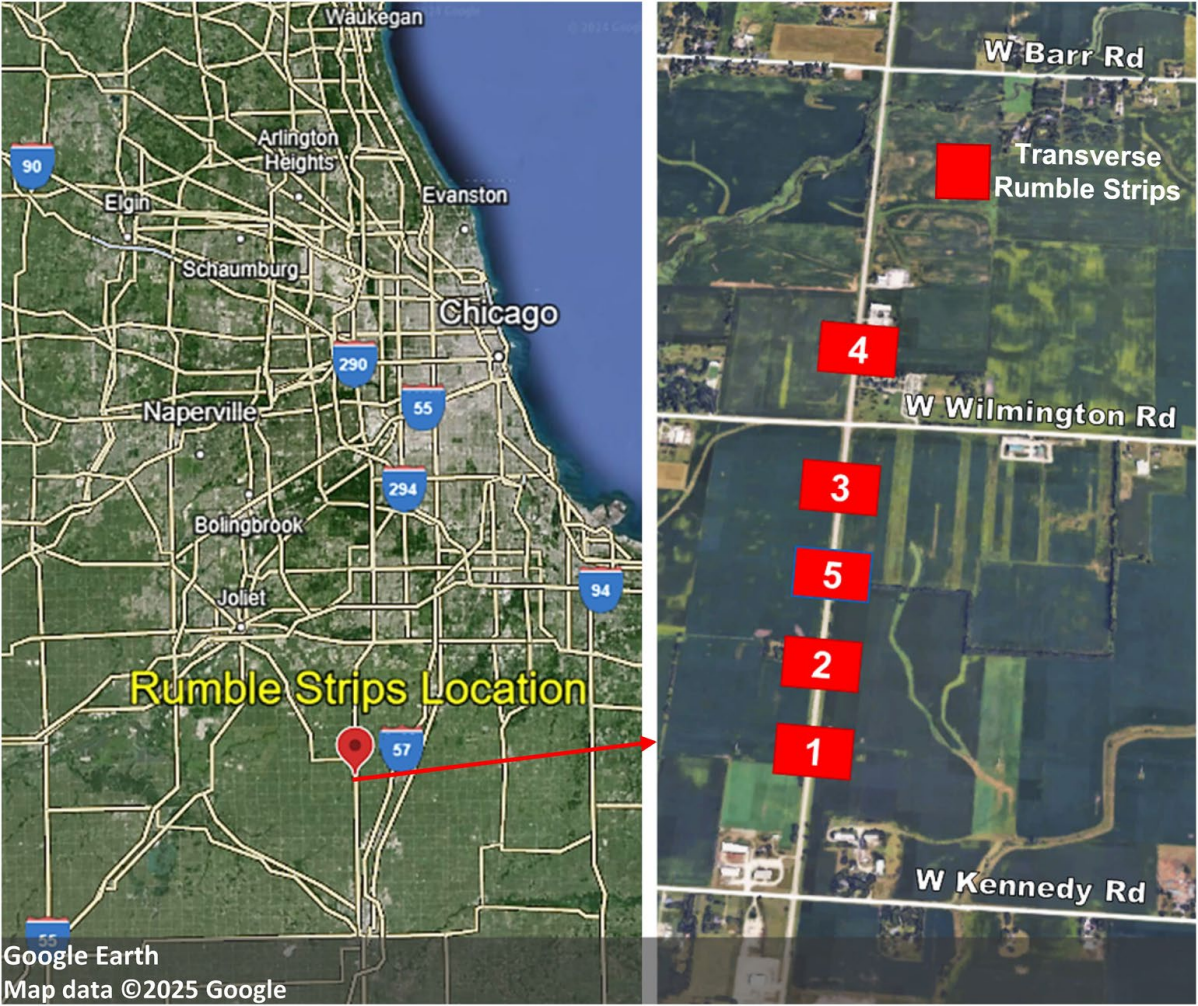
Location of Transverse rumble strips on US-45.

The five transverse rumble strip designs were milled on US-45 in Illinois between Kennedy and Barr Roads, as illustrated in Fig. 4. The location of these five designs was selected to meet the requirements of the AASHTO Statistical Isolated Pass-by (SIP) method^28^, which specifies that the testing location should be: (1) straight with a uniform flat pavement to minimize the effect of any fluctuations in the asphalt pavement surface on collected noise data; (2) clear of debris, gravel, and sound barriers such as signboards to avoid blocking the normal propagation of sound waves and interference with noise data; and (3) far from major noise sources such as railway stations and airports to prevent contaminating the collected noise data.

**Fig. 5:**
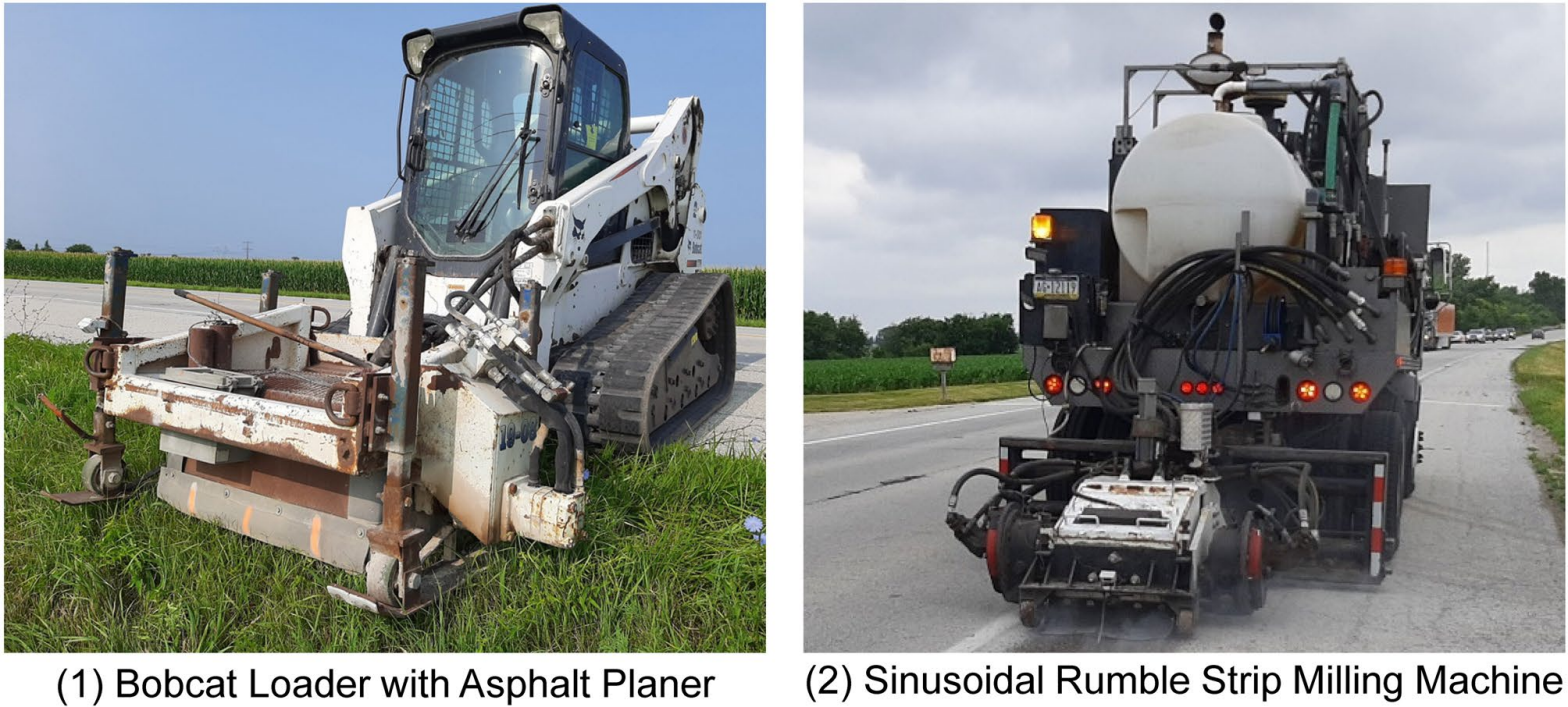
Milling equipment for traditional and sinusoidal transverse designs.

**Fig. 6:**
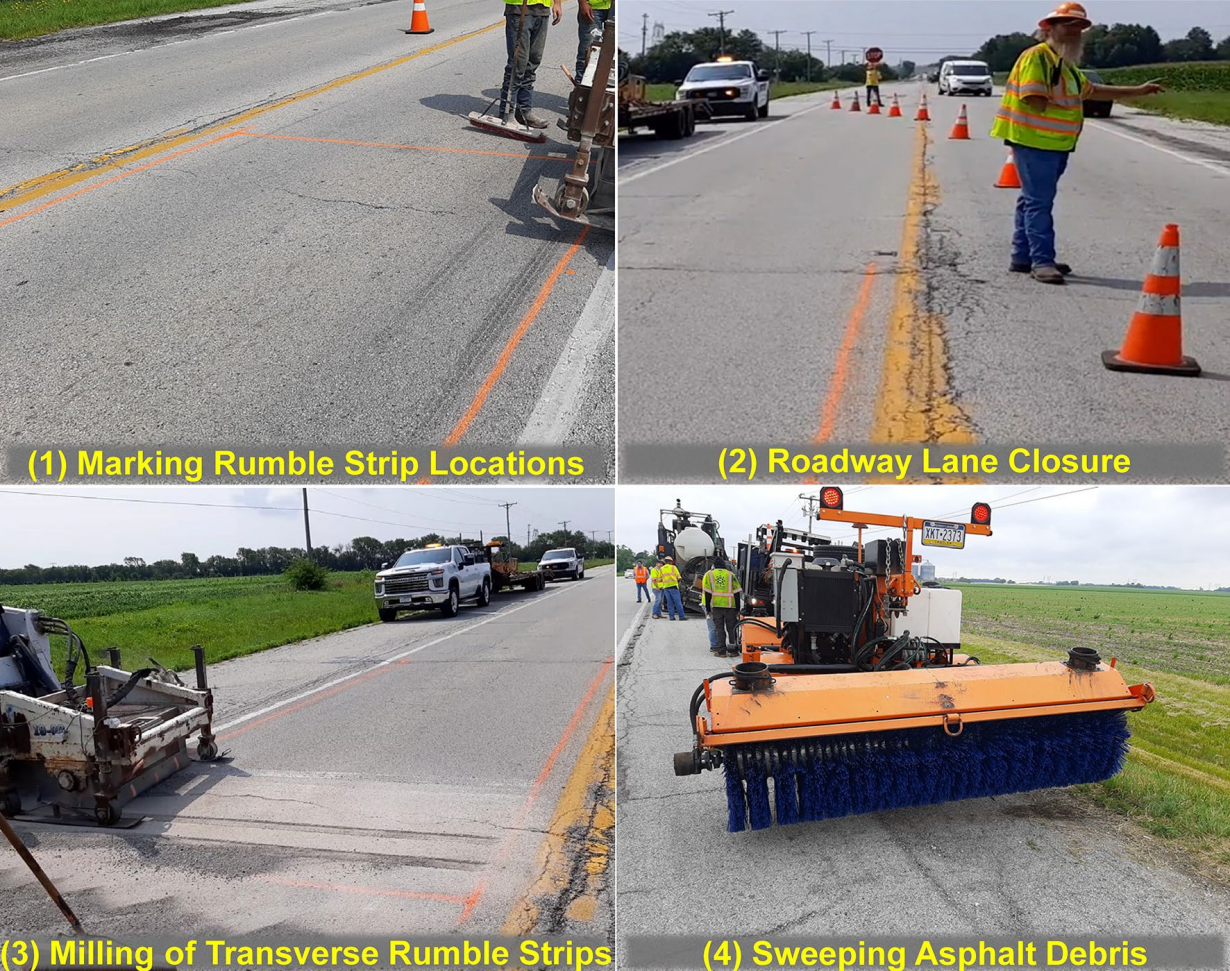
Milling sequence of transverse rumble strips.

These transverse rumble strip designs were constructed by a contractor who used a bobcat loader with an asphalt planer to mill the traditional designs (Designs 1, 2, 3, and 4) and a computer-controlled milling machine to construct the sinusoidal design (Design 5), as illustrated in Fig. 5. Therefore, the construction cost of sinusoidal rumble strip designs is often reported to be slightly higher than that of traditional designs due to the use of computer-controlled milling equipment^29^. The milling of transverse rumble strips was implemented in four consecutive steps: (1) labeling the positions of the transverse rumble strip designs on the roadway following the planned layout, (2) closing the roadway lane to oncoming traffic, (3) milling the transverse rumble strips, and (4) sweeping the road surface to clean all asphalt debris resulting from the rumble strip milling, as illustrated in Fig. 6.

### Setup of testing equipment and collection of noise measurements

**Fig. 7:**
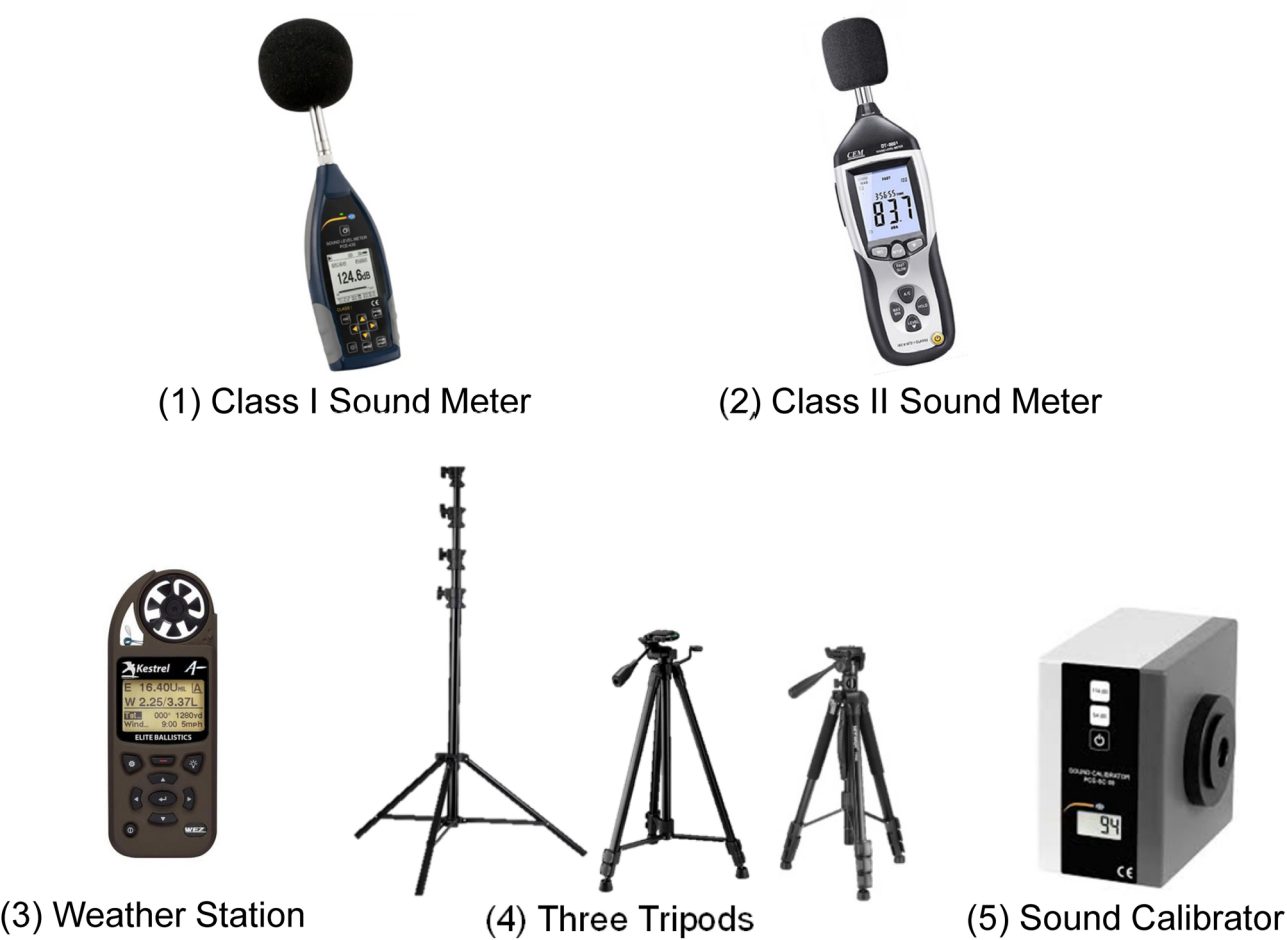
Noise gathering equipment.

The noise testing equipment was installed to gather in-vehicle and external noise levels generated by the five transverse rumble strip designs in accordance with the SAE J1477 standard for measuring internal noise levels^30^ and the AASHTO Statistical Isolated Pass-by (SIP) method (AASHTO T 389 − 20, 2020), respectively. These noise measurements were gathered utilizing (1) two class I sound meters for external noise measurements, (2) one class II sound meter for in-vehicle noise measurements, (3) a weather station, (4) three tripods, and (5) a sound calibrator, as shown in Fig. 7.

#### Equipment installation for gathering in-vehicle noise levels

**Fig. 8:**
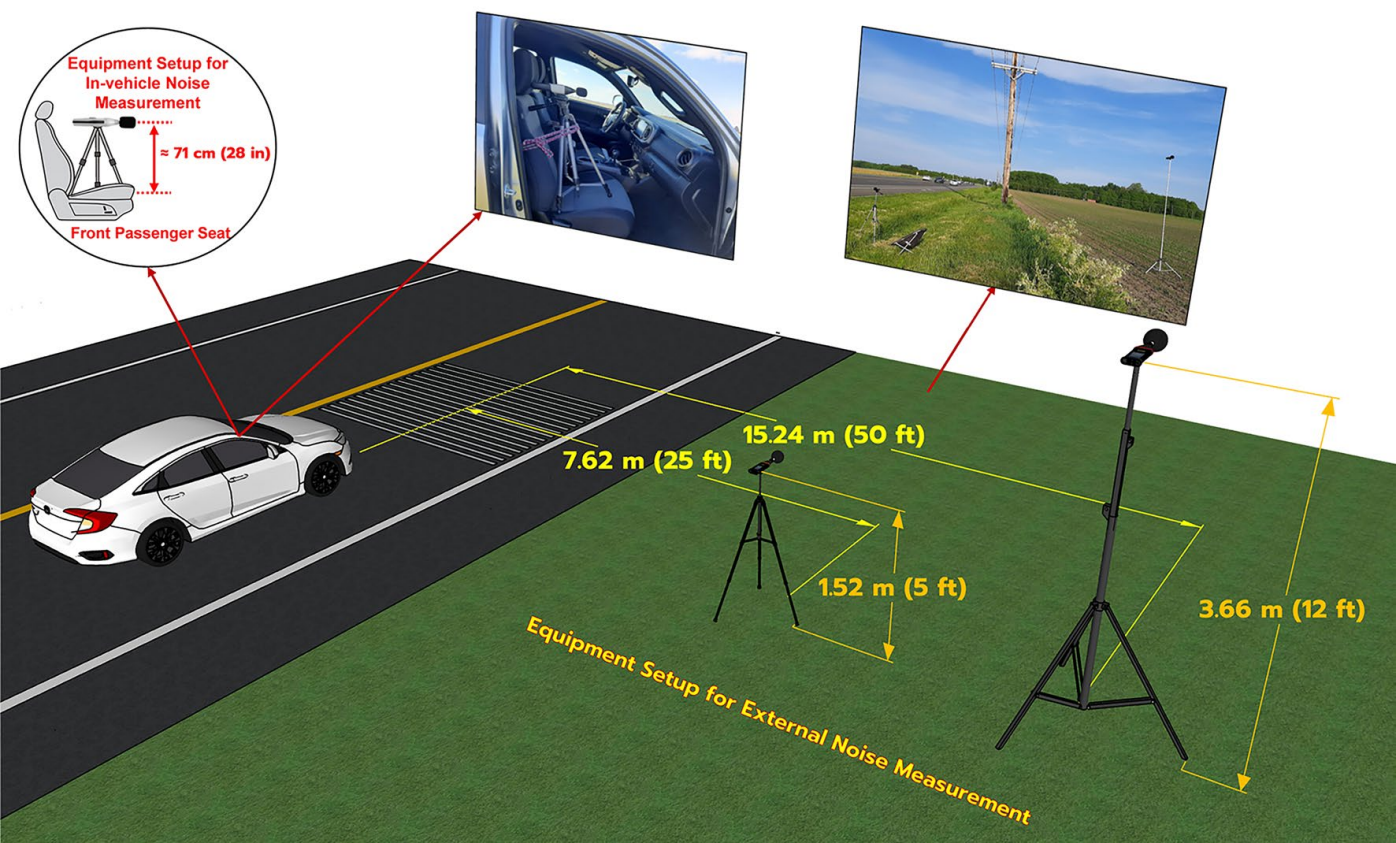
Equipment configuration for collecting noise measurements.

The measurement equipment for in-vehicle noise was set up according to the SAE standard J1477 to gather both the roadway baseline and rumble strip strike noise levels inside the vehicle. The measurement equipment for in-vehicle noise is composed of one class II sound meter that was firmly fixed on a tripod at 71 cm ± 5 cm (28 in ± 2 in) above the vehicle’s front passenger seat while being pointed towards the traveling direction^30^, as shown in Fig. 8.

#### Equipment installation for gathering external noise levels

The equipment for gathering external noise measurements was installed according to the AASHTO SIP method to collect the external baseline and rumble strip strike noise levels. This equipment included two class I sound meters that were installed on two tripods at heights of 1.52 m (5 ft) and 3.66 m (12 ft) above the roadway level and placed at 7.62 m (25 ft) and 15.24 m (50 ft) from the center of the travel lane, respectively, (AASHTO T 389 − 20, 2020), as illustrated in Fig. 8.

#### Collection of in-vehicle and external noise measurements

**Fig. 9:**
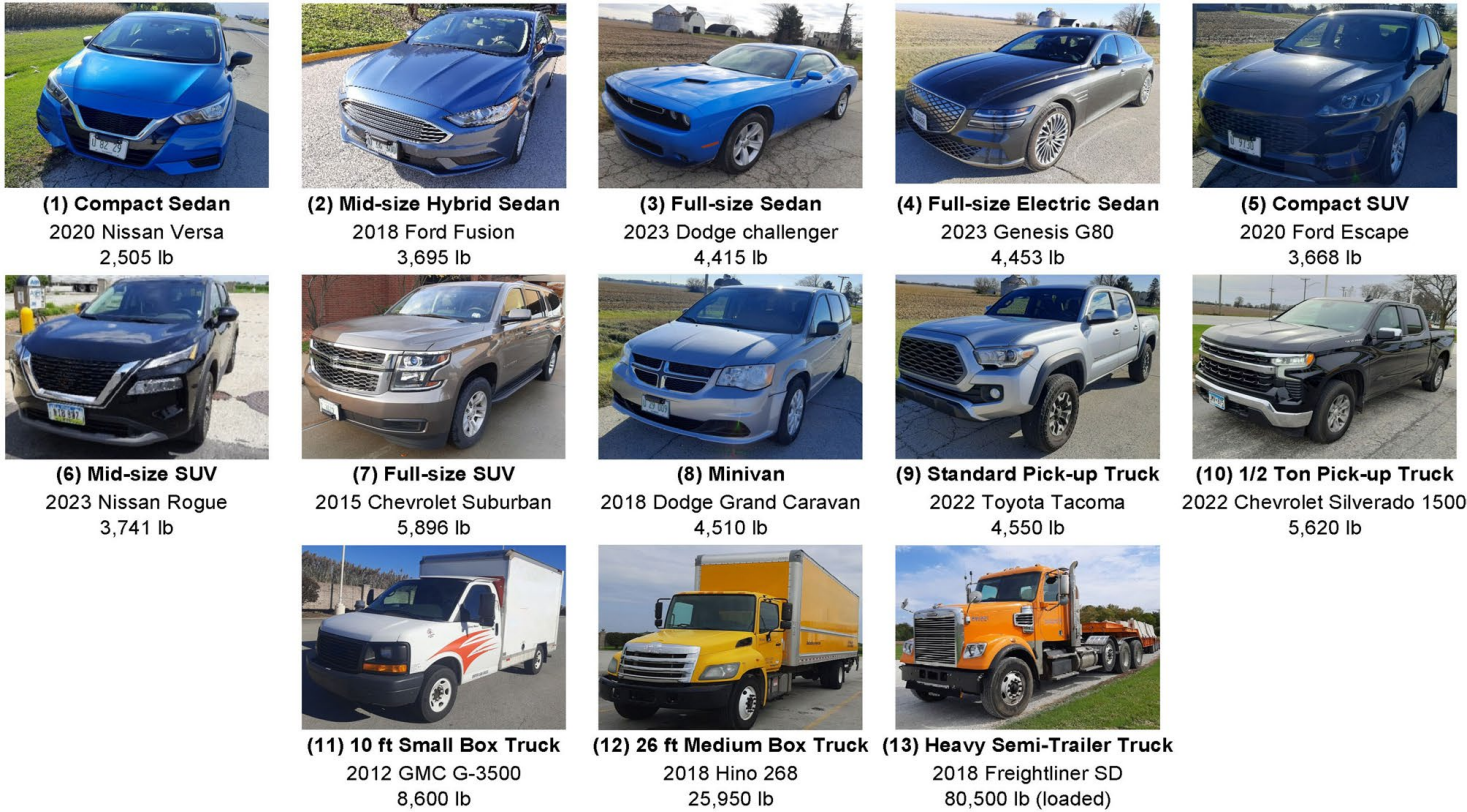
Sizes, types, models, makes, and weights of test vehicles utilized in field testing.

The in-vehicle and external noise levels produced by the five transverse rumble strip designs were collected using thirteen test vehicles that consisted of sedans, SUVs, minivan, pick-up trucks, box trucks, and heavy semi-trailer truck with gasoline, electric, and hybrid engines, as illustrated in Fig. 9. These thirteen test vehicles were carefully selected with sufficient variations in vehicle type, make, size, weight, engine, and model to provide a comprehensive representation of all vehicles on U.S. roadways. All thirteen test vehicles were utilized to strike each of the five transverse rumble strip designs while traveling at a speed of 80.5 kph (50 mph). For each transverse rumble design, the in-vehicle and external noise levels were gathered by conducting at least (a) six valid strikes by the sedan cars, SUVs, minivan, pick-up trucks, and small box truck; and (b) four valid strikes by the medium box truck and heavy semi-trailer truck because it was challenging to maneuver these large vehicles on a two-lane undivided roadway. The in-vehicle noise measurements were collected every 0.5 s while turning off A/C, windshield wipers, signal flashers, car windows, and any potential source of in-vehicle noise to prevent contaminating the collected noise data^30^. Similarly, the external noise measurements produced by each test vehicle were collected only when there were no additional noise levels including those generated by nearby vehicles to prevent the contamination of collected noise measurements. Vehicle passes were only considered valid when no other vehicles or extraneous noise sources were present during the collection of external noise data. Any test pass affected by nearby traffic was excluded from the dataset and repeated to ensure data integrity. Additionally, the site weather conditions during the external noise measurements including wind speed and air temperature were recorded during the measurements to ensure dry roadway conditions and wind speed does not exceed the AASHTO SIP method-specified maximum threshold of 17.7 kph (11 mph) (AASHTO T 389 − 20, 2020). The air temperature ranged from 18.3 to 31 ˚C (65 to 88 ˚F) and the wind speed ranged from 4.8 to 16 kph (3 to 10 mph).

### Analysis of collected in-vehicle and external noise measurements

**Fig. 10:**
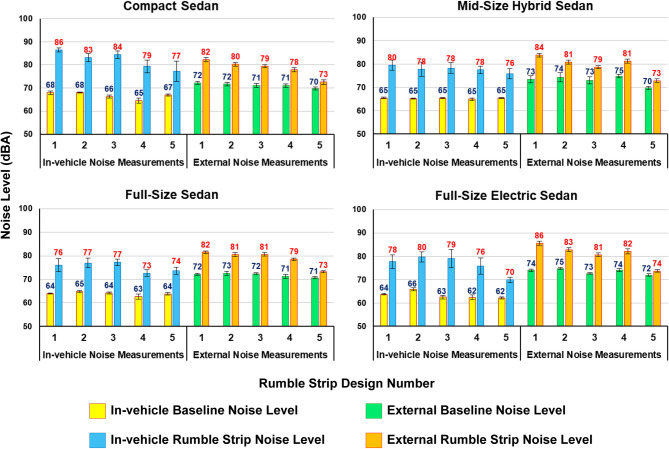
In-Vehicle and external noise levels generated by sedan cars.

**Fig. 11:**
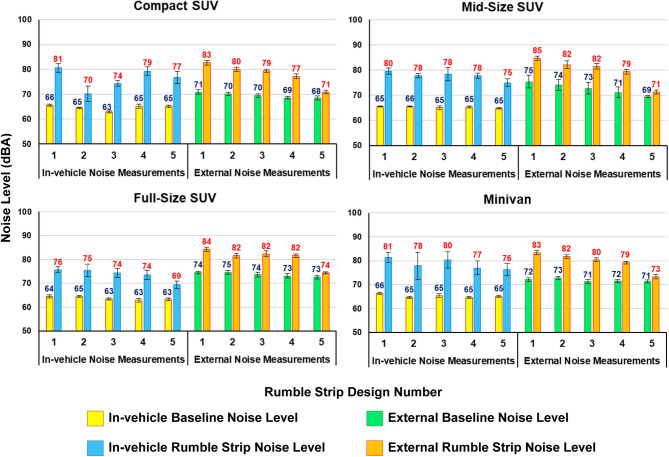
In-Vehicle and external noise levels generated by SUVs and minivan.

**Fig. 12:**
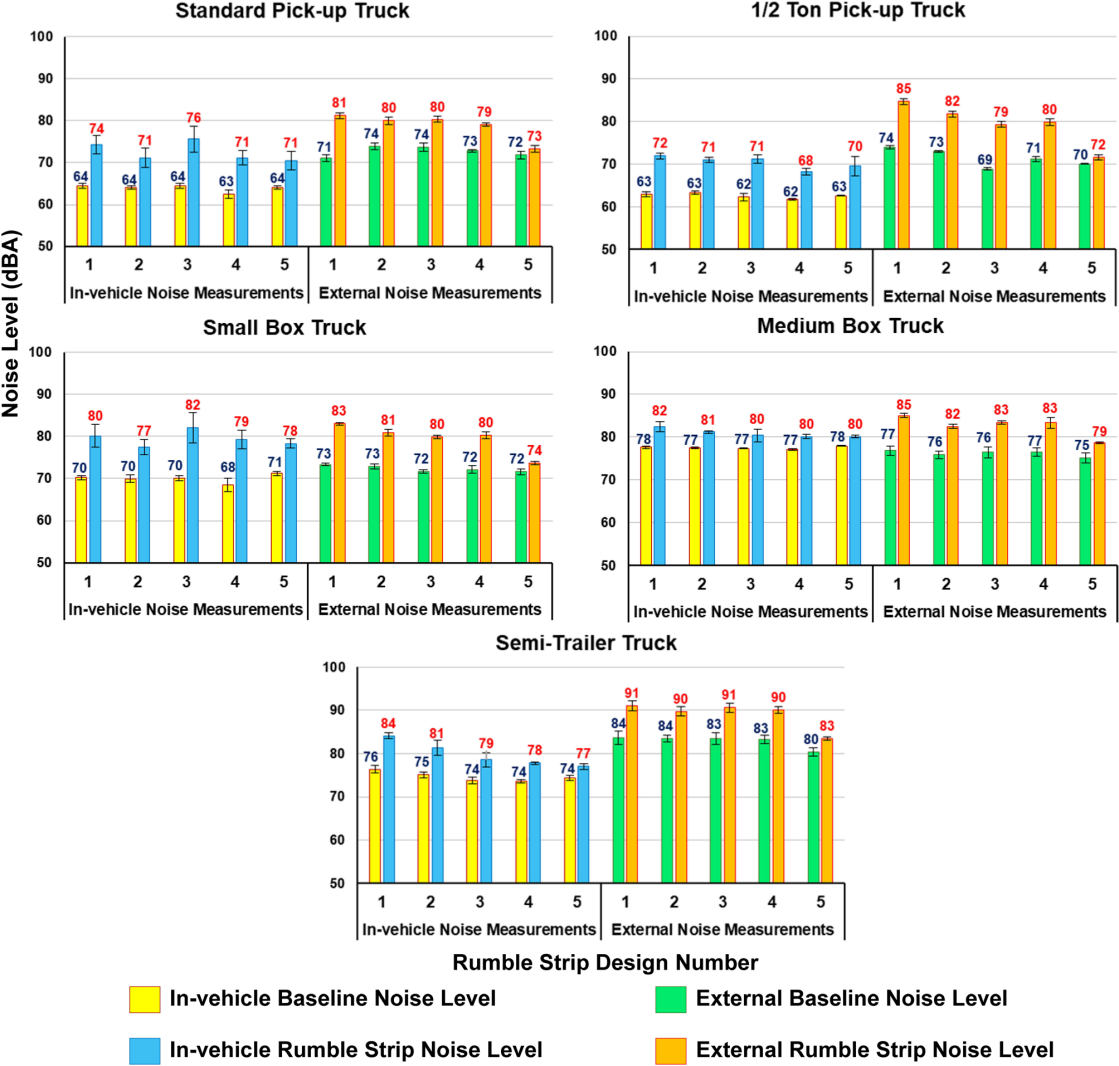
In-vehicle and external noise levels generated by trucks.

**Fig. 13:**
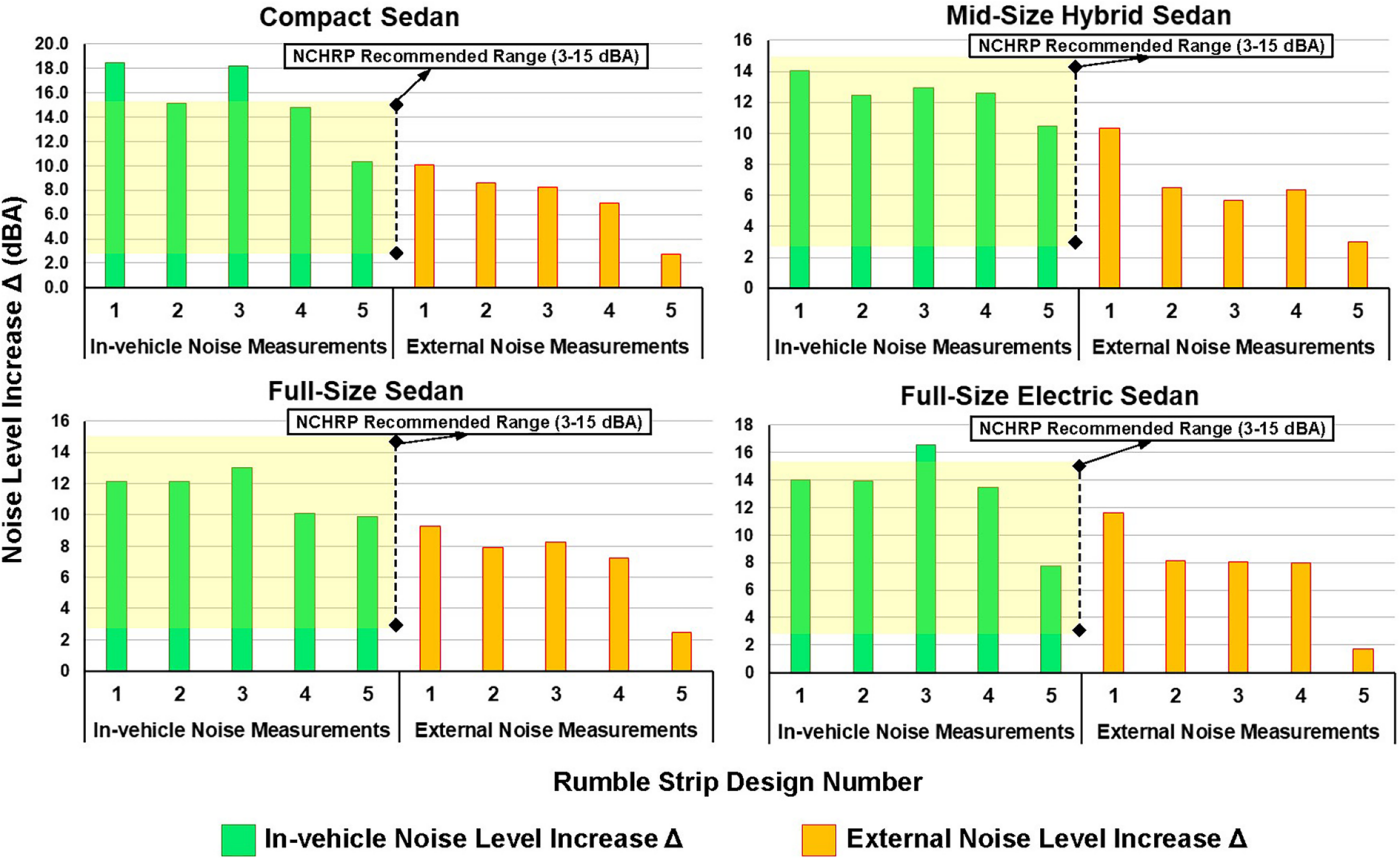
In-vehicle and external noise levels noise level increases generated by sedan cars.

**Fig. 14:**
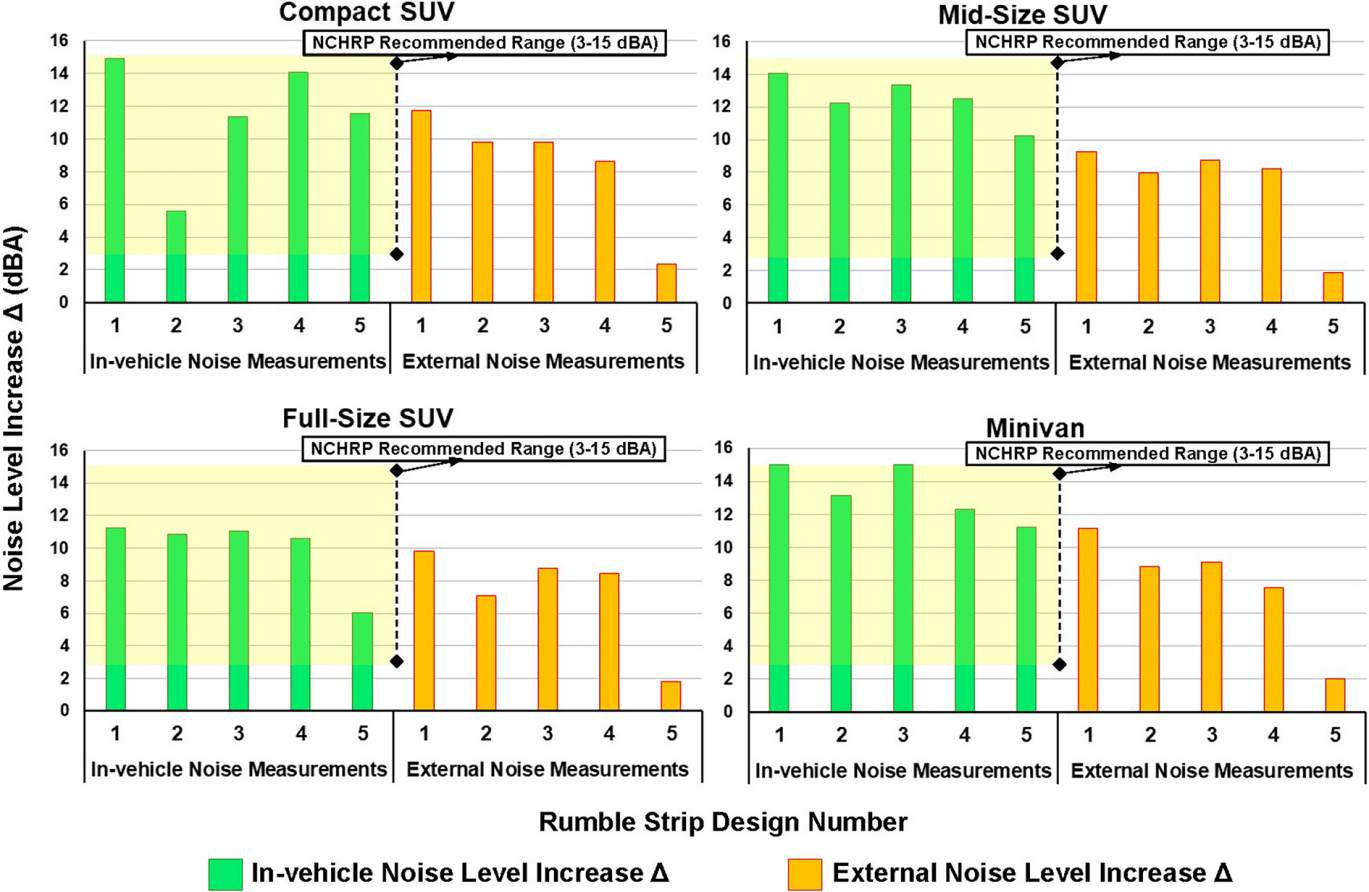
In-vehicle and external noise level increases generated by SUVs and minivan.

**Fig. 15:**
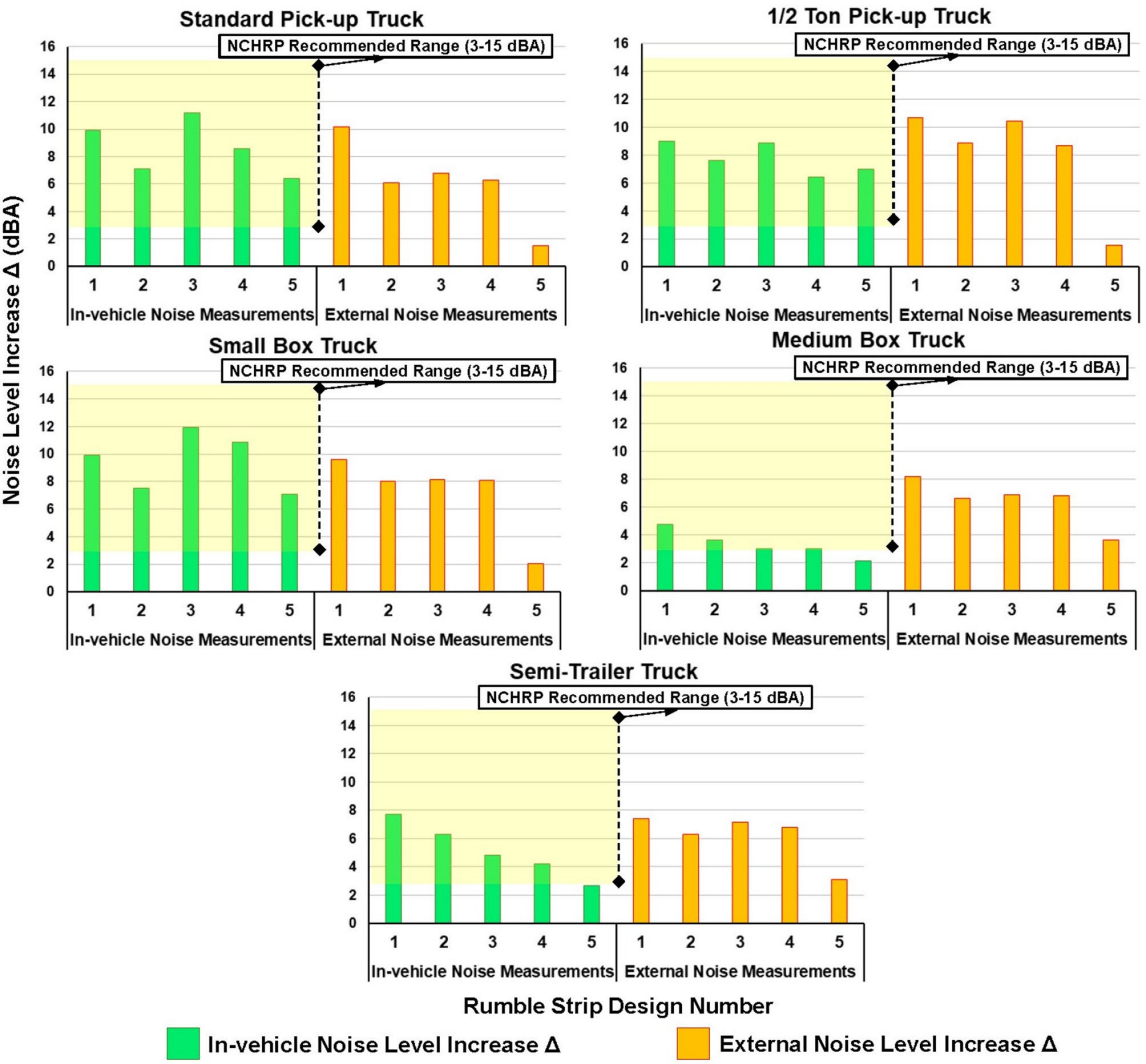
In-vehicle and external noise level increases generated by trucks.

The in-vehicle and external noise measurements generated by the five transverse rumble strip designs were analyzed for each pair of rumble strip design (i) and test vehicle (j) to calculate: (1) average baseline in-vehicle and external noise levels (BLN_ij_) produced by each test vehicle while traveling over the road immediately before hitting the rumble strips, and (2) average rumble strip in-vehicle and external noise levels (RSN_ij_) produced by each test vehicle traveling over each design of the five rumble strips, as illustrated in Figs. 10 and 11, and 12. These noise levels were then utilized to compute average in-vehicle and external noise level increases (Δ_ij_) produced by test vehicle (j) striking each rumble strip design (i) using Eq. (1), as illustrated in Figs. 13 and 14, and 15.

1 $$\Delta _{{ij}} = RSN_{{ij}} - {\text{ }}BLN_{{ij}}$$

These in-vehicle and external noise level increases (Δ_ij_) were then evaluated to identify top-performing transverse rumble strip designs that produce in-vehicle noise increases between 3 and 15 dBA, as recommended by the NCHRP to alert distracted drivers^15^ while reducing roadside noise and related complaints from nearby residents. This was achieved in three steps that focused on analyzing in-vehicle noise level increases, evaluating external noise level increases, and identifying top-performing transverse rumble strip designs. The following sections describe these three analysis steps.

#### Analysis of in-vehicle noise measurements

The in-vehicle noise level increases (Δin_ij_) for each pair of rumble strip design (i) and test vehicle (j) were averaged for all thirteen test vehicles (j = 1 to 13) to calculate an overall average in-vehicle noise level increase (Δin_i_) for each rumble strip design (i) using Eq. (2), as illustrated in Table 1. These calculated overall average in-vehicle noise level increases were utilized to evaluate the performance of the tested transverse rumble strip designs in generating in-vehicle noise level increases between 3 and 15 dBA that satisfy the NCHRP recommendations^15^,. The results of this evaluation showed that all the tested transverse rumble strip designs produced overall average in-vehicle noise level increases for the thirteen test vehicles (Δin_i_) that meet the NCHRP recommendations, as presented in Table 1. It should be noted, however, that the generated in-vehicle noise level increases (Δin_ij_) by designs 1, 2, and 3 for two of the test vehicles exceeded the maximum NCHRP recommended limit of 15 dBA while that of design 5 was slightly lower than the minimum NCHRP recommended limit of 3 dBA for two other test vehicles, as shown in Table 1. The analysis results showed that traditional transverse rumble strip design 1 produced in-vehicle noise level increase (Δin_1−1_) of 18.4 dBA for the tested compact sedan (j = 1). Similarly, shorter traditional transverse rumble strip design 2 generated in-vehicle level increase (Δin_2−1_) of 15.2 dBA for the tested compact sedan (j = 1). Angled traditional transverse rumble strip design 3 generated in-vehicle level increases (Δin_3−j_) of 18.2 dBA and 16.5 dBA for the compact sedan (j = 1) and the full-size electric sedan (j = 4), respectively. On the other hand, sinusoidal transverse rumble strip design 5 produced in-vehicle level increases (Δin_5−j_) of 2.2 dBA and 2.7 dBA for the medium box truck (j = 12) and the semi-trailer truck (j = 13), respectively, as presented in Table 1.

2 $$\Delta {\mathrm{in}}_{{\mathrm{i}}} {\text{ = }}\frac{{\sum {_{{{\text{j = 1}}}}^{{{\text{j = 13}}}} } \Delta {\mathrm{in}}_{{{\mathrm{ij}}}} }}{{{\mathrm{13}}}}$$

**Table 1:** In-Vehicle Noise Level Increases Produced by Transverse Rumble Strip Designs.

Test vehicle (j)	In-vehicle noise increase Δin_ij_ (dBA) and rumble strip design (i)				
	1	2	3	4	5
1. Compact sedan	18.4*	15.2*	18.2*	14.8	10.4
2. Mid-size hybrid sedan	14.1	12.5	12.9	12.6	10.5
3. Full-size sedan	12.2	12.1	13.0	10.1	9.9
4. Full-size electric sedan	14.0	13.9	16.5*	13.5	7.7
5. Compact SUV	14.9	5.6	11.4	14.1	11.6
6. Mid-size SUV	14.1	12.2	13.3	12.5	10.2
7. Full-size SUV	11.2	10.9	11.0	10.6	6.0
8. Minivan	15.0	13.1	15.0	12.3	11.2
9. Standard pick-up truck	9.9	7.1	11.2	8.6	6.4
10. 1/2 Ton pick-up truck	9.0	7.6	8.9	6.5	7.0
11. Small box truck	9.9	7.5	11.9	10.8	7.1
12. Medium box truck	4.8	3.6	3.0	3.0	2.2**
13. Semi-trailer truck	7.7	6.3	4.8	4.2	2.7**
**Overall average Δin**_**i**_ **(dBA)**	**11.9**	**9.8**	**11.6**	**10.3**	**7.9**
**Standard deviation (dBA)**	**3.5**	**3.5**	**4.0**	**3.6**	**3.0**

**Overall average Δin**_**i**_ is the mean in-vehicle noise level increase for each rumble strip design (i = 1 to 5) and all thirteen test vehicles (j = 1 to 13).

**Standard deviation** is the standard deviation of the in-vehicle noise level increases for each rumble strip design (i = 1 to 5) and all thirteen test vehicles (j = 1 to 13).

* *In-vehicle noise level increases that exceed recommended NCHRP limit of 15 dBA*.

** *In-vehicle noise level increases that are lower than recommended NCHRP limit of 3 dBA*.

#### Analysis of External noise measurements

To identify transverse rumble strip designs that minimize roadside noise, the external noise level increases (Δex_ij_) for each pair of rumble strip design (i) and test vehicle (j) were averaged for all thirteen test vehicles (j = 1 to 13) to calculate an overall average external noise level increase (Δex_i_) for each rumble strip design (i) using Eq. (3), as presented in Table 2.

3 $$\Delta {\mathrm{ex}}_{{\mathrm{i}}} {\text{ = }}\frac{{\sum {_{{{\text{j = 1}}}}^{{{\text{j = 13}}}} } \Delta {\mathrm{ex}}_{{{\mathrm{ij}}}} }}{{{\mathrm{13}}}}$$

The calculated overall average external noise level increases (Δex_i_) of the four transverse rumble strip designs (i = 2 to 5) were then compared with the overall average external noise level increase of the baseline design 1. The comparison indicated that sinusoidal design 5, staggered traditional design 4, shorter traditional design 2, and angled traditional design 3 reduced the roadside noise levels by 77% (7.7 dBA), 24% (2.4 dBA), 22% (2.2 dBA), and 18% (1.8 dBA), respectively, compared to baseline traditional design 1, as presented in Table 2. These findings were verified using a paired t-test that was performed for the external noise level increases (Δex_ij_) generated by each transverse rumble strip design and baseline traditional design 1 using a significance level (α) of 0.05, a confidence level of 95%, and 12 degrees of freedom, as shown in Table 2. This statistical test was performed to determine if a rumble strip design caused a significant difference in the generated overall average external noise level increase (Δex_i_) compared to baseline traditional design 1. The analysis results confirmed that the four tested transverse rumble strip designs (designs 2, 3, 4, and 5) generated statistically significant reductions in their average external noise level increases compared to baseline traditional shoulder design 1 (t-statistic > t-critical = 2.178, p < α = 0.05), as shown in Table 2. Additionally, one-way repeated-measures ANOVA test was conducted to simultaneously analyze and compare the impact of the five tested transverse rumble strip designs on the generated external noise level increases while accounting for variability within test vehicles. The analysis results confirmed a statistically significant effect of each of the five tested transverse rumble strip designs on generated external noise level increases (F-statistic = 133.7 > F-critical = 2.565, *p* = 2.2E-25 < α = 0.05), as shown in Table 2. Furthermore, 95% confidence intervals were calculated for the mean noise reductions using vehicle-level paired differences in noise levels between each rumble strip design and baseline design 1. These confidence intervals provided an estimate of the precision of the noise reduction effects achieved by the tested transverse rumble strip designs and showed the stability of these effects across the thirteen test vehicles, as shown in Table 2.

**Table 2:** External noise level increases produced by transverse rumble strip designs.

Test vehicle (j)		External noise increase Δex_ij_ (dBA) and rumble strip design (i)				
		1	2	3	4	5
1. Compact sedan		10.1	8.6	8.2	7.0	2.8
2. Mid-size hybrid sedan		10.4	6.5	5.6	6.4	3.0
3. Full-size sedan		9.3	8.0	8.3	7.2	2.5
4. Full-size electric sedan		11.6	8.1	8.1	8.0	1.7
5. Compact SUV		11.8	9.8	9.8	8.6	2.4
6. Mid-size SUV		9.2	8.0	8.8	8.2	1.9
7. Full-size SUV		9.8	7.1	8.8	8.4	1.8
8. Minivan		11.2	8.8	9.1	7.6	2.0
9. Standard pick-up truck		10.2	6.1	6.8	6.3	1.5
10. 1/2 Ton pick-up truck		10.7	8.9	10.4	8.7	1.5
11. Small box truck		9.6	8.0	8.2	8.1	2.1
12. Medium box truck		8.2	6.6	6.9	6.8	3.6
13. Semi-trailer truck		7.4	6.3	7.1	6.8	3.1
t-statistic*		**Baseline**	**7.7**	**4.8**	**7.3**	**16.1**
p-value*		**Baseline**	**5.2E-06**	**4.7E-04**	**9.5E-06**	**1.8E-09**
F-statistic**		**133.7**				
p-value**		**2.2E-25**				
**Standard Deviation (dBA)**		**1.2**	**1.1**	**1.3**	**0.8**	**0.6**
**Overall Average Δex**_**i**_ **(dBA)**		**9.9**	**7.7**	**8.2**	**7.5**	**2.3**
**Average Noise Reduction (dBA)**		**Baseline**	**-2.2**	**-1.8**	**-2.4**	**-7.7**
**Average Noise Reduction (%)**		**Baseline**	**-22%**	**-18%**	**-24%**	**-77%**
95% Confidence Interval for Noise Reduction (dBA)***	**Upper CI**	**Baseline**	**-1.6**	**-1.0**	**-1.7**	**-6.6**
	**Lower CI**	**Baseline**	**-2.8**	**-2.6**	**-3.1**	**-8.7**

t-statistic* and p-value*are results of a paired t-test that was performed for the external noise level increases (Δex_ij_) generated by each transverse rumble strip design and baseline traditional design 1 to determine if a rumble strip design caused a significant difference in the generated overall average external noise level increase (Δex_i_) compared to the baseline traditional design.

F-statistic** and p-value** are results of a one-way repeated-measures ANOVA test that was conducted to simultaneously analyze and compare the impact of the five tested transverse rumble strip designs on the generated external noise level increases while accounting for variability within test vehicles.

**Standard Deviation** is the standard deviation of the external noise level increases for each rumble strip design (i = 1 to 5) and all thirteen test vehicles (j = 1 to 13).

**Average Noise Reduction** is the mean reduction in external noise achieved by each rumble strip design (i = 2 to 5) compared to the baseline traditional design (i = 1).

**95% Confidence Interval for Noise Reduction** is calculated for the mean noise reductions using vehicle-level paired differences in noise levels between each rumble strip design and baseline design 1 to provided an estimate of the precision and stability of the noise reduction effects achieved by the rumble strip designs (i = 2 to 5) across the thirteen test vehicles.

*Paired t-test: *t-critical = 2.178*,* Significance Level (α) = 0.05*,* Degrees of Freedom (df) = 12*

**One-way Repeated-Measures ANOVA: F-critical = 2.565, Significance Level (α) = 0.05, Degrees of Freedom (df) = 4, Error = 48

***Confidence Intervals (CI): *calculated using vehicle-level paired difference in noise levels between each rumble strip design and baseline design 1.*

#### Discussion of analysis results

Baseline traditional design 1 and shorter traditional design 2 generated average external noise level increases of 9.9 and 7.7 dBA, respectively. These values are consistent with the external noise increase ranges reported in previous studies that evaluated comparable traditional transverse rumble strip designs with rectangular cross sections^12,27^. For example, An et al.^27^, reported that the external noise level increases generated by three traditional transverse rumble strip designs with rectangular cross sections ranged from 5 to 18 dBA. Similarly, Horne et al.^12^, tested two comparable rectangular transverse rumble strip designs and reported that their external noise level increase ranged from 5 to 11 dBA. The tested shorter traditional design 2 in this study reduced generated roadside noise levels by 2.2 dBA compared to baseline traditional design 1. These findings are consistent with those provided by Hallmark et al.^6^, who reported a reduction in external noise levels of 2.6 dBA achieved by a 6-transverse rumble strip panel compared to a 12-strip panel.

**Fig. 16:**
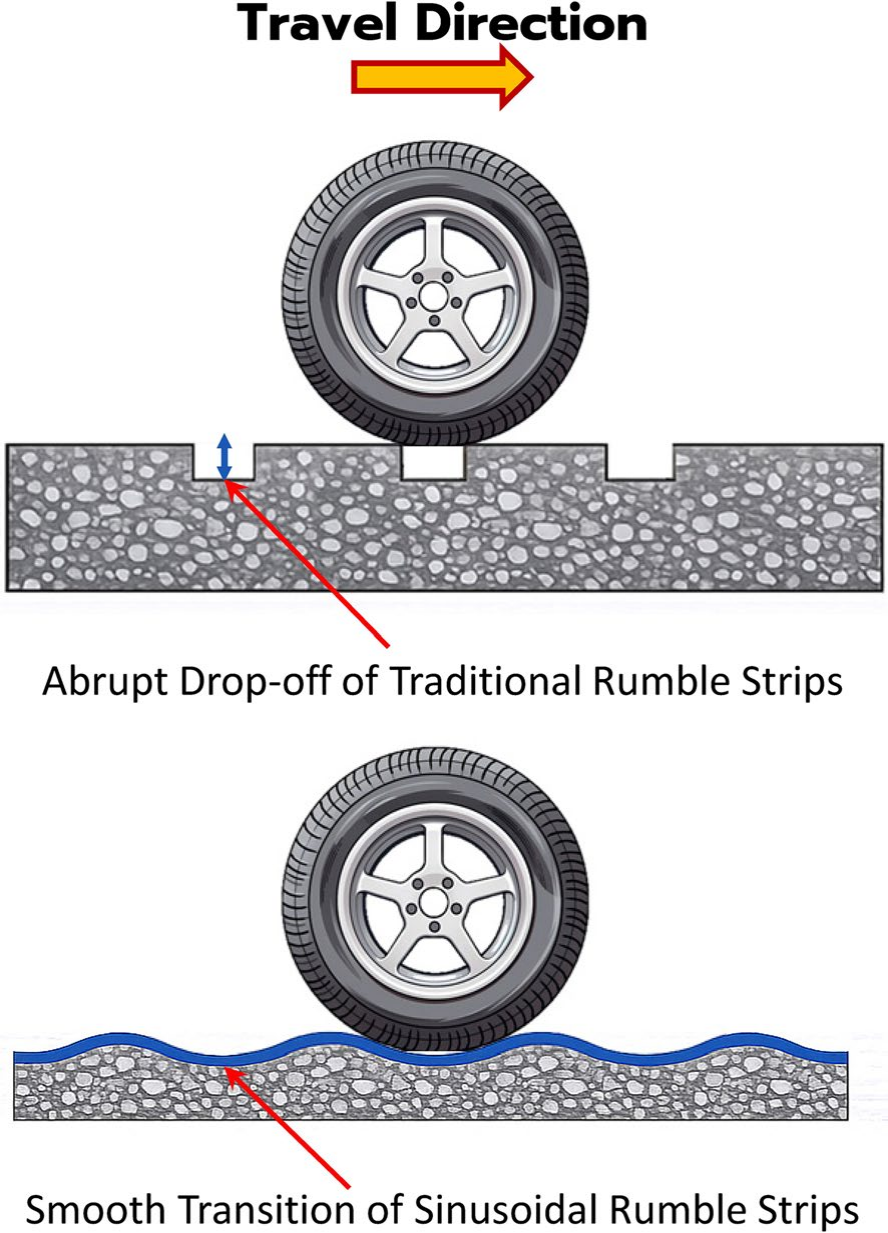
Transition of tires over traditional and sinusoidal transverse rumble strips.

Traditional transverse rumble strip designs have sharp-edged rectangular grooves that cause abrupt drop-off and rapid transition between the vehicle tires and the rumble strip grooves, as shown in Fig. 16. This abrupt drop-off generates high-magnitude impact forces at the tire–pavement interface, which in turn produce relatively high in-vehicle and roadside noise levels. Similar observations have been reported in prior studies that attributed high noise levels of traditional rumble strips to their discontinuous geometry and sharp transitions^19,31^. On the other hand, the sinusoidal transverse rumble strip design uses a continuous, smooth wavelength profile that causes a gradual and smooth transition of the tires rather than producing discrete impact events, as shown in Fig. 16^31^. This gradual and smooth transition of tires reduces the impact forces generated at the tire–pavement interface resulting in lower in-vehicle and roadside noise levels. This explains the significant roadside noise reduction of 7.7 dBA (77%) generated by the sinusoidal design in this study compared to the baseline traditional design while still maintaining adequate in-vehicle noise levels to alert distracted drivers.

**Fig. 17:**
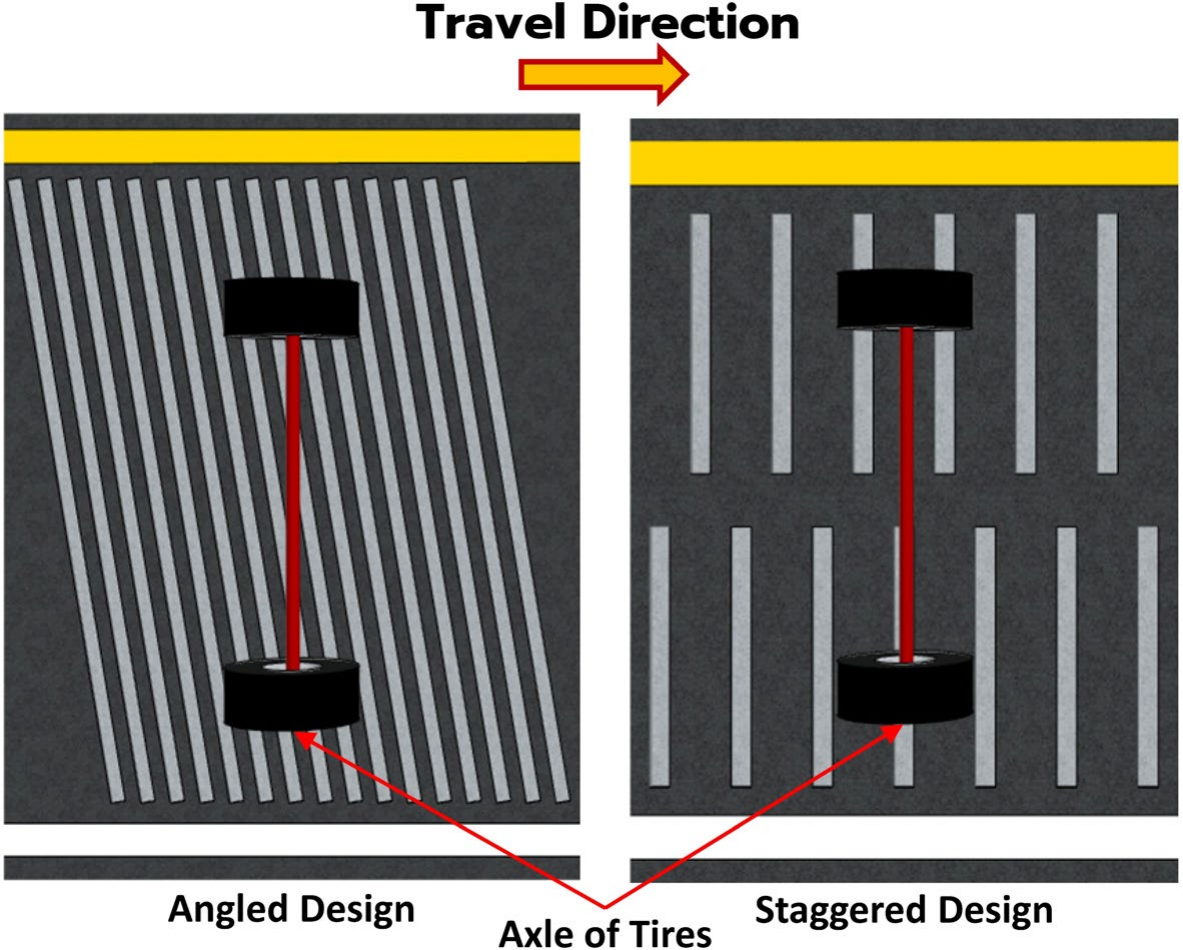
Interaction between axle of tires angled and staggered transverse rumble strips.

The intermediate performance of the shorter panel, angled, and staggered designs further supports this physical interpretation. The shorter panel design allows for a fewer number of interactions between tire and rumble strip grooves due to its fewer number of transverse strips per panel. This results in less generated impact forces leading to lower roadside and in-vehicle noise levels compared to the baseline traditional design. Additionally, the angled and staggered designs partially disrupt the continuity of tire impacts. The inclined and staggered strips prevent the simultaneous interaction between each axle’s tires (right and left tires) and the grooves of rumble strips, as shown in Fig. 17. This minimizes the magnitude of generated impact forces at the tire–pavement interface causing a reduction in roadside and in-vehicle noise. It should be noted that the reductions in roadside noise achieved by these three designs are less than those caused by the sinusoidal design because they still utilize the traditional sharp-edged rectangular grooves.

#### Top-performing transverse rumble strip designs

**Fig. 18:**
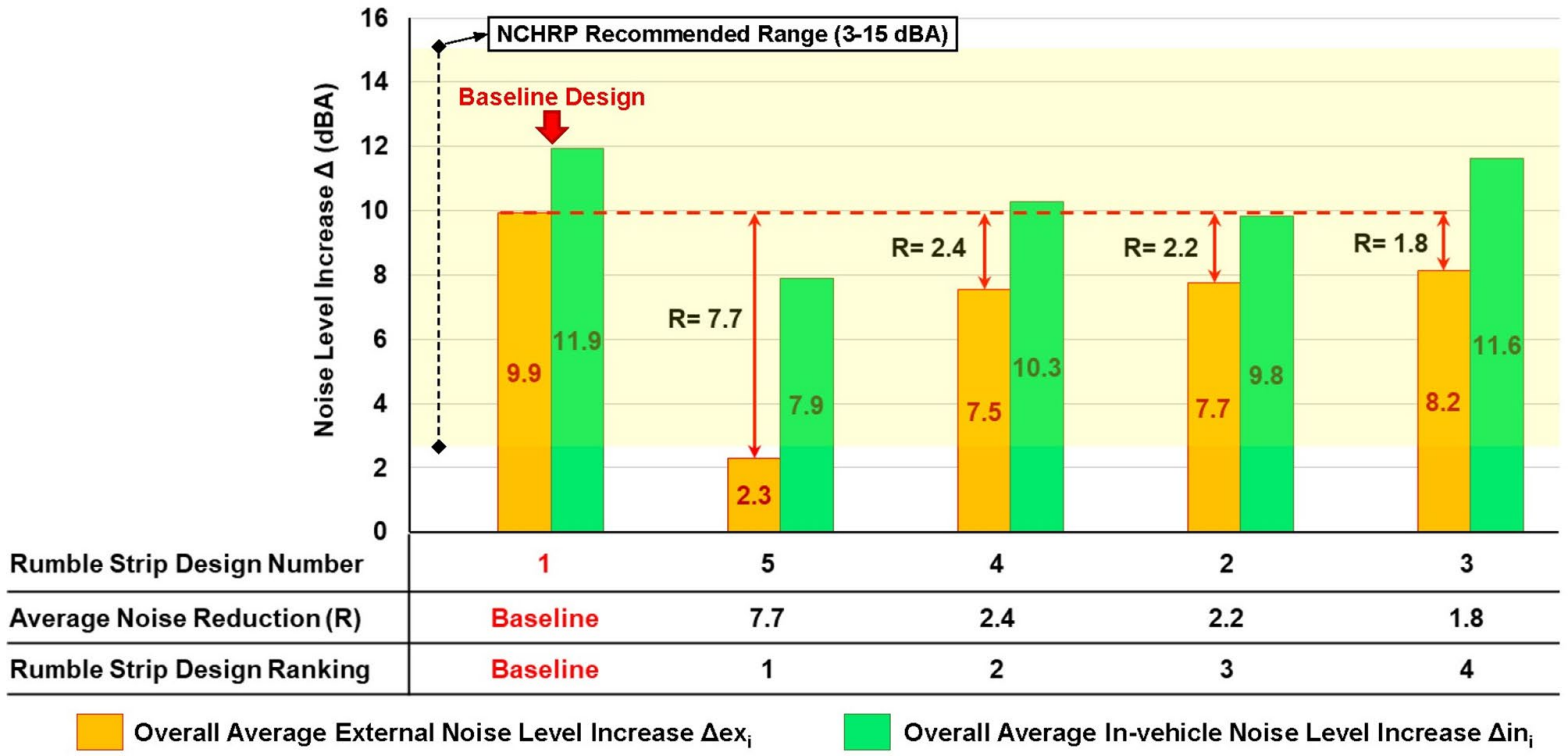
Top-performing transverse rumble strip designs.

The overall average in-vehicle and external noise level increases of the five transverse rumble strip designs were analyzed to identify top-performing designs and rank them based on their ability to minimize roadside noise levels while meeting the NCHRP recommendations of generating in-vehicle noise level increases between 3 and 15 dBA for alerting distracted drivers. All the five transverse rumble strip designs satisfied the NCHRP recommendations for in-vehicle noise level increases. Accordingly, they were ranked based on their average reduction in roadside noise (R) compared to baseline traditional design 1, as illustrated in Fig. 18. The top-ranked transverse rumble strip design was sinusoidal design 5 that achieved an average reduction in roadside noise of 7.7 dBA followed by designs 4 and 2 that achieved an average reduction in roadside noise by 2.4 dBA and 2.2 dBA, respectively, as shown in Fig. 18. On the other hand, angled traditional design 3 made the least reduction in roadside noise of 1.8 dBA and scored the lowest rank among the modified and alternative designs, as illustrated in Fig. 18. Accordingly, sinusoidal design 5 provides a promising transverse rumble strip design that can maintain roadway safety benefits for road users while minimizing roadside noise and potential complaints from nearby residents. It should be noted that these rankings are based on the noise performance criterion and do not consider other criteria that might be important to transportation agencies such as construction and maintenance costs. As discussed earlier in Sect. 2.1, the construction cost of the four traditional designs (Designs 1, 2, 3, and 4) are similar since they were all constructed in this study using a bobcat loader with an asphalt planer, as shown in Fig. 5. On the other hand, the construction cost of sinusoidal rumble strip designs is often reported to be slightly higher than that of traditional designs due to the use of computer-controlled milling machine^29^.

Despite the aforementioned finding that sinusoidal design 5 provided the highest reduction in roadside noise, its effectiveness in alerting the drivers of heavy trucks was more limited compared to the other tested designs. This sinusoidal design generated average in-vehicle noise level increases for the medium box truck and semi-trailer truck that were slightly less than the NCHRP minimum threshold of 3 dBA recommended for alerting inattentive drivers (see Table 1). Accordingly, this sinusoidal design cannot be universally recommended for all roadway types. For example, this sinusoidal design can be utilized on roadways that are heavily used by passenger vehicles and light trucks to minimize roadside noise and potential complaints from nearby residents based on the conditions evaluated in this study. For other roadways with a significant volume of heavy trucks, other transverse rumble strip designs such as staggered, shorter panel, and angled designs can be more effective in providing a more balanced performance for alerting drivers of heavy trucks while minimizing roadside noise levels.

## Limitations and future research

This study was conducted under controlled field conditions to investigate the effect of varying transverse rumble strip designs on generated in-vehicle and roadside noise levels. All noise measurements were collected at a travel speed of 80.5 kph (50 mph) because it was the posted speed limit on the study roadway where the field measurements were conducted. This allowed for collecting maximum potential noise levels generated by vehicles traveling at the maximum speed limit while maintaining consistency across all test passes. It should be noted that vehicle speed has been reported to directly influence the generated roadside noise with higher speeds producing higher noise levels^32^. Accordingly, future research can expand the findings of this study by collecting and analyzing in-vehicle and roadside noise levels generated by vehicles with a wider range of travel speeds.

In addition, all measurements were performed on a single uniform asphalt pavement surface in accordance with the SAE J1477 standard and the AASHTO Statistical Isolated Pass-by (SIP) method. While this approach ensured measurement consistency and enabled meaningful comparisons among rumble strip designs, the findings may not be directly generalizable to other pavement types and surface textures. Changes in pavement properties may affect the absolute noise levels generated by tested rumble strip designs. Despite this limitation, the findings of this study in terms of the performance ranking of tested designs are still applicable to other pavement types and surfaces. Accordingly, the noise levels presented in this study should be interpreted as performance comparisons of different transverse rumble strip designs under controlled conditions specific to this pavement surface. Therefore, future research is recommended to examine the performance of these designs on different pavement surfaces to further evaluate their noise performance.

Furthermore, this study utilized thirteen different test vehicles that provide a representative sample of all vehicle types on U.S. roadways to ensure a comprehensive evaluation of the performance of the five tested transverse rumble strip designs. It should be noted, however, that this study did not analyze the individual influence of each vehicle class such sedans, SUVs, and trucks on generated in-vehicle and roadside noise. Future studies can conduct detailed analyses to provide a better understanding of transverse rumble strip design behavior across vehicle classes. Moreover, the noise measurements were collected during dry pavement conditions in accordance with SAE J1477 and the AASHTO Statistical Isolated Pass-by (SIP) method. The effects of varying weather conditions, such as wet pavement, precipitation, or temperature extremes, on the roadside and in-vehicle noise generated by transverse rumble strip designs were not evaluated in this study and are recommended for future investigation. Additionally, tire parameters such as tread pattern, aspect ratio, rim size, compound stiffness, and inflation pressure were not individually controlled or evaluated. Variability in these tire parameters may influence the generated noise levels. Therefore, future research is recommended to isolate and quantify the individual effect of each tire parameter on the roadside and in-vehicle noise levels generated by transverse rumble strip designs.

## Conclusion

A comprehensive field study was conducted to evaluate the in-vehicle and external noise performance of five promising traditional and alternative transverse rumble strip designs in generating effective in-vehicle noise level increases to alert distracted and drowsy drivers while reducing roadside noise levels. The first phase of this study focused on identifying and constructing five transverse rumble strip designs on US-45 in Illinois. The constructed five transverse rumble strip designs consisted of one baseline traditional design that was obtained from IDOT local roads and streets design manual; two modified traditional designs that provide new design dimensions and parameters including shorter panel and angled transverse rumble strips; and two alternative designs that provide original geometric patterns including staggered and sinusoidal transverse rumble strips. The second phase of study focused on collecting in-vehicle and external noise measurements using thirteen different vehicles that were carefully selected with sufficient variations in vehicle type, make, size, weight, engine, and model to represent all types of vehicles on U.S. roads. The in-vehicle and external field measurements were conducted in accordance with the SAE J1477 standard and the AASHTO Statistical Isolated Pass-by (SIP) method, respectively. The third phase focused on analyzing the collected in-vehicle and external noise measurements of the five transverse rumble strip designs to evaluate their in-vehicle and external noise performance. The analysis results confirmed that the five tested transverse rumble strip designs generated overall in-vehicle noise level increases between 3 and 15 dBA which fulfill the NCHRP recommendations for alerting distracted or drowsy drivers. The analysis results also indicated that sinusoidal design 5, staggered traditional design 4, shorter traditional design 2, and angled traditional design 3 reduced the roadside noise levels by 77% (7.7 dBA), 24% (2.4 dBA), 22% (2.2 dBA), and 18% (1.8 dBA), respectively, compared to baseline traditional design 1. These four promising transverse rumble strip designs were capable of alerting distracted drivers while decreasing roadside noise levels under the tested conditions in this study. These findings are expected to help state DOTs select and construct effective transverse rumble strip designs that maintain roadway safety while minimizing noise impact on nearby residents.

## Supplementary Information

Below is the link to the electronic supplementary material.

Supplementary Material 1

## Data Availability

Data will be made available on request.
