# Supplementary material for: Evaluating the effectiveness and roadside noise of alternative transverse rumble strip designs
Source: Sci Rep. 2026 May 19;16:21627. doi: 10.1038/s41598-026-48504-4 (PMC13354790; doi:10.1038/s41598-026-48504-4)

Objective

- Conduct field tests to evaluate performance of five transverse rumble strip designs in maintaining NCHRP recommended in-vehicle noise level increases to alert distracted drivers while reducing roadside noise.

Methodology

- Construction of five transverse rumble strip designs on US-45 in Illinois.
- Field measurements of in-vehicle and external noise levels following SAE J1477 standard and AASHTO SIP using 13 test vehicles that represent all vehicle types on U.S. roadways.

Conclusion

- Four novel designs including shorter panel, angled, staggered, and sinusoidal transverse rumble strip designs:
  - ✓ Generated adequate in-vehicle noise to alert distracted drivers.
  - ✓ Reduced roadside noise levels by a range of 18% to 77%.

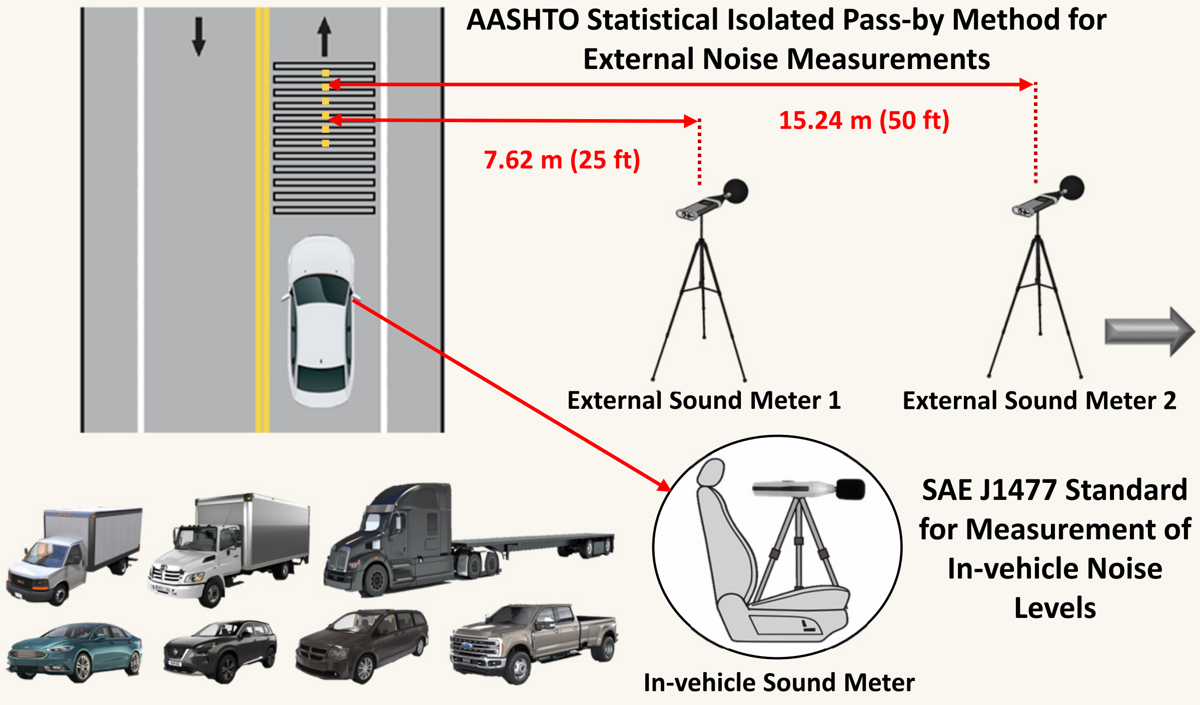

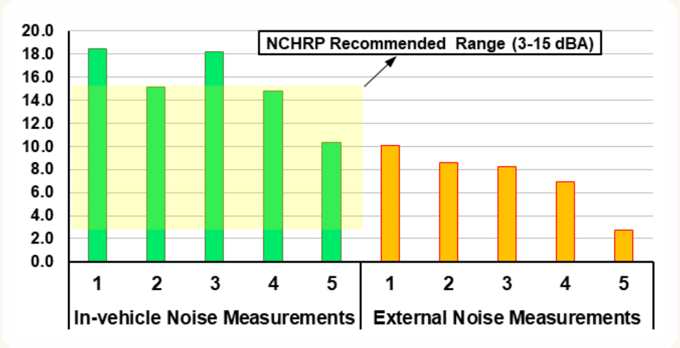

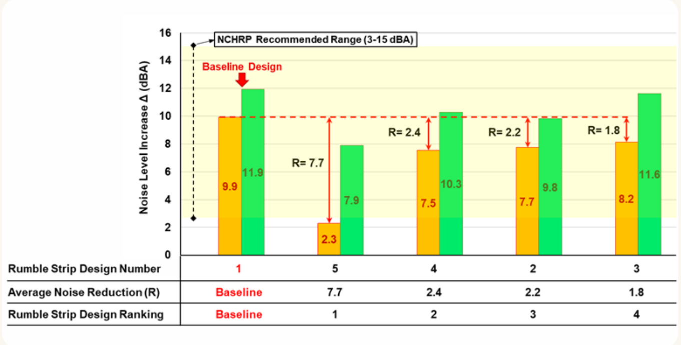

Supplement: Supplementary file 1 — Supplementary Material 1 [file 41598_2026_48504_MOESM1_ESM.pdf]
